# Diverse drug delivery systems for the enhancement of cancer immunotherapy: an overview

**DOI:** 10.3389/fimmu.2024.1328145

**Published:** 2024-01-17

**Authors:** Xu Liu, Yang Cheng, Yao Mu, Zhaohan Zhang, Dan Tian, Yunpeng Liu, Xuejun Hu, Ti Wen

**Affiliations:** ^1^ Department of Respiratory and Infectious Disease of Geriatrics, The First Hospital of China Medical University, Shenyang, Liaoning, China; ^2^ No.20 High School, Shenyang, Liaoning, China; ^3^ Department of Thoracic Surgery, Guangdong Provincial People’s Hospital, Guangdong Academy of Medical Sciences, Guangzhou, China; ^4^ Key Laboratory of Anticancer Drugs and Biotherapy of Liaoning Province, The First Hospital of China Medical University, Shenyang, Liaoning, China; ^5^ Department of Medical Oncology, The First Hospital of China Medical University, Shenyang, Liaoning, China; ^6^ Liaoning Province Clinical Research Center for Cancer, The First Hospital of China Medical University, Shenyang, Liaoning, China; ^7^ Clinical Cancer Treatment and Research Center of Shenyang, The First Hospital of China Medical University, Shenyang, Liaoning, China

**Keywords:** cancer immunotherapy, drug delivery, nanoparticles, coupled drugs, protein degradation, multidrug combination

## Abstract

Despite the clear benefits demonstrated by immunotherapy, there is still an inevitable off-target effect resulting in serious adverse immune reactions. In recent years, the research and development of Drug Delivery System (DDS) has received increased prominence. In decades of development, DDS has demonstrated the ability to deliver drugs in a precisely targeted manner to mitigate side effects and has the advantages of flexible control of drug release, improved pharmacokinetics, and drug distribution. Therefore, we consider that combining cancer immunotherapy with DDS can enhance the anti-tumor ability. In this paper, we provide an overview of the latest drug delivery strategies in cancer immunotherapy and briefly introduce the characteristics of DDS based on nano-carriers (liposomes, polymer nano-micelles, mesoporous silica, extracellular vesicles, etc.) and coupling technology (ADCs, PDCs and targeted protein degradation). Our aim is to show readers a variety of drug delivery platforms under different immune mechanisms, and analyze their advantages and limitations, to provide more superior and accurate targeting strategies for cancer immunotherapy.

## 1 Introduction

Cancer is a major global public health problem that poses a severe threat to human health and is increasing in incidence and mortality (1). Immunotherapy prevents, controls, and eliminates cancer by exogenously interfering with the body’s immune system to enhance or restore the body’s ability to fight tumors. Compared with traditional therapy, immunotherapy has become a promising alternative therapy for various malignant tumors, including cytokines immune checkpoint inhibitors (ICIs), cancer vaccines and so on, showing remarkable clinical results (1–4). Despite the clear benefits demonstrated by immunotherapy, there are still several issues that urgently need to be addressed in clinical application. Immunotherapy depends on systemic injection of immunological drugs (such as monoclonal antibodies). Still, this approach tends to distribute the drugs to various tissues and organs throughout the body, failing to achieve precise targeting of the lesions and thus causing a series of immune-related adverse reactions (4–6).

While developing effective anti-tumor immunological drugs, issues that can optimize the therapeutic effects should be given attention, including how to improve the pharmacokinetics and *in vivo* distribution of the drugs, to increase the specificity of the targeted cells and the intracellular drug accumulation, as well as to reduce the systemic severe side-effects arising from the non-specific reactions of the drugs. Consequently, the research and development of drug delivery systems (DDS) is increasingly emphasized (7–9). Based on various chemical or biomaterials as carriers for drug delivery, or by coupling ligands targeted to specific cells with drugs, DDS has the advantages of controlling drug release, improving drug solubility, improving pharmacokinetics and drug distribution (9–11). In addition, the surface modification of drug carriers (such as nanoparticles) with targeted ligands can accurately deliver drugs to the target site, to improve drug efficacy and reduce adverse reactions (12). Hence, the collaboration of tumor immunotherapy and DDS can deliver immunological drugs to specific sites precisely and efficiently, thus achieving practical anti-tumor effects (13).

**Figure 1:**
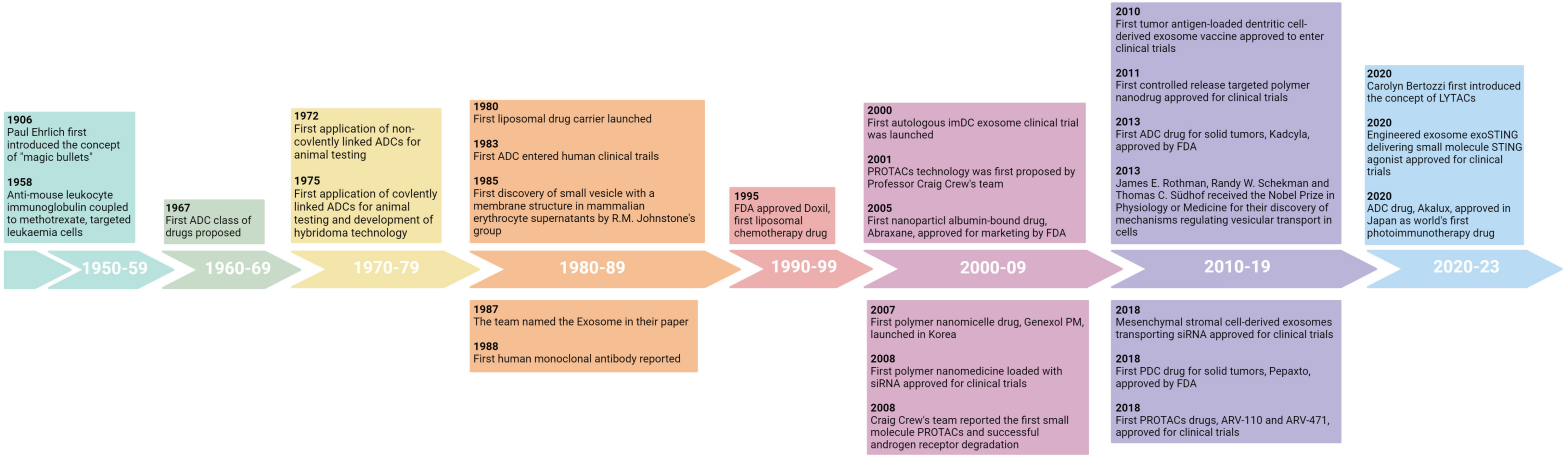
The evolution of drug delivery systems for cancer therapy.

DDS originated in the early 20th century when Professor Paul Ehrlich developed the concept of “magic bullets”, in which cytotoxic drugs were mounted on specific monoclonal antibodies to kill tumor cells (14). Decades later, antibody-drug conjugates (ADCs) were introduced (14). In recent decades, with the maturity of pharmaceutical technology, nanoparticles such as liposomes, polymer nanoparticles, extracellular vesicles and various coupling drugs have been used in clinic (**Figure 1**). So far, dozens of nanoparticles and coupling drugs have been approved for cancer treatment, as shown in **Tables 1**, **2** (14, 15). But most of them are used to deliver cytotoxic drugs for chemotherapy. With the continuous development of novel delivery platforms, such as extracellular vesicles (EVs), biomimetic nanoparticles, virus-like particles (VLPs), hydrogels, *etc.*, more and more studies are being conducted on the use of poorly stabilized and patterned drugs (*e.g.*, proteins, peptides, antibodies, and nucleic acids) for tumor immunotherapy (13, 16–19). Continuously improved delivery technology not only enables safer and more controllable effective targeting of immunomodulators to the desired tumor or immune cells, but also provides a platform for multi-drug combinations. In this paper, we introduce DDS from the perspectives of nanoparticle-based drug delivery system and coupling technology and review the latest research on their application in tumor immunotherapy.

**Table 1:** Nanocarrier drugs that have been approved for the treatment of solid tumors (up to April 2023).

Name	Particle type/drug	Company	Application	Date of first approval
Doxil	Liposome Doxorubicin	Janssen	Ovarian cancer, HIV‐associated Kaposi’s sarcoma	1995
DaunoXome	Liposome Daunorubicin	Galen	HIV‐associated Kaposi’s sarcoma	1996
Myocet	Liposome Doxorubicin	Teva UK	Metastatic breast cancer	2000
Abraxane	Nanoparticle albumin-bound Paclitaxel	Celgene	Breast cancer, advanced nonsmall cell lung cancer (NSCLC), metastatic pancreatic cancer	2005
Genexol PM	Polymer micelle Paclitaxel	Samyang Biopharm	Metastatic breast cancer, NSCLC, ovarian cancer	2007
Mepact	Liposome Mifamurtide	Millennium	Osteosarcoma	2009
Nanoxel M	Polymer micelle Docetaxel	Samyang Biopharm	Metastatic esophageal squamous cell carcinoma	2012
Onivyde	Liposome Irinotecan	Merrimack	Metastatic pancreatic ductal adenocarcinoma	2015
Apealea	Polymer micelles Paclitaxel	Elevar Therapeutics	Epithelial ovarian cancer, primary peritoneal cancer, fallopian tube cancer	2018
Hensify	Hafnium oxide nanoparticles Radiotherapy	Nanobiotix	Locally advanced squamous cell carcinoma of the head and neck	2019
Fyarro	Nanoparticle albumin-bound Sirolimus	Aadi Bioscience	Metastatic perivascular epithelioid cell tumor	2021

**Table 2:** ADCs that have been approved for treatment of solid tumors (up to April 2023).

Name	Drug(antibody/payload)	Target	Application	Date of first approval
Kadcyla	Trastuzumab/DM1	HER2	HER2 positive breast cancer	2013
Enhertu	Trastuzumab/Dxd	HER2	HER2 positive breast cancer, HER2 positive gastric cancer	2019
Padcev	Enfortumab/MMAE	Nectin-4	Urothelial cancer	2019
Trodelvy	Sacituzumab/SN38	Trop-2	Triple-negative breast cancer (TNBC)	2020
AKalux	Cetuximab/IRDye700DX	EGFR	Head and neck cancer	2020
Tivdak	Tisotumab/MMAE	TF	Cervical cancer	2021
Aidixi	Hertuzumab/MMAE	HER2	HER2 over-expression gastric cancer	2021
Elahere	Mirvetuximab/DM4	FR-α	Ovarian cancer	2022

## 2 DDS based on nanoparticles for cancer immunotherapy

**Figure 2:**
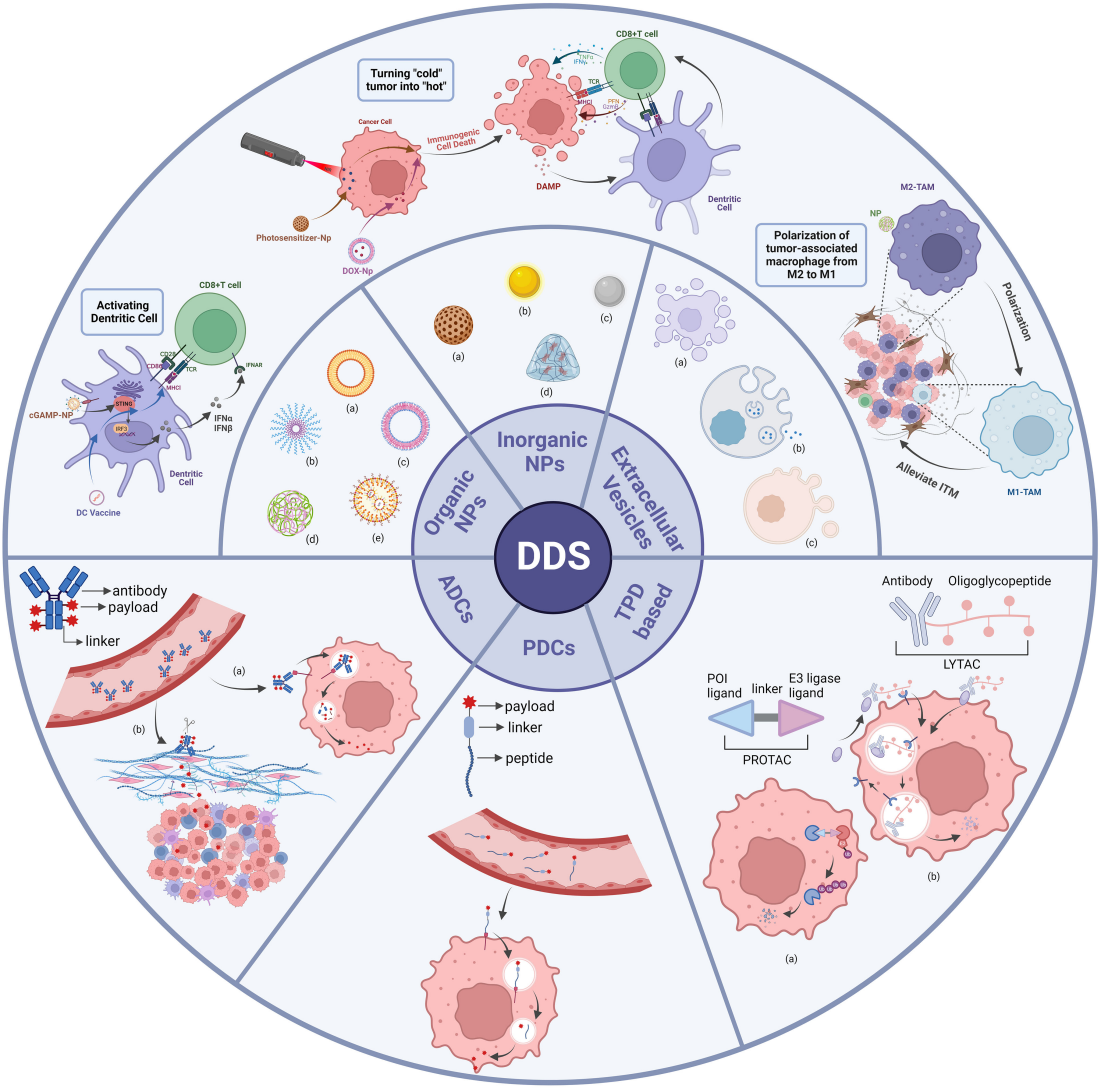
Classes of DDS. Organic NPs, inorganic NPs and extracellular vesicles are categorized as subclasses of nanoparticles-based DDS. Nanoparticles that enhance anti-tumor immune responses through different mechanisms are shown here. ADCs, PDCs and TPD are categorized as subclasses of coupling technology-based DDS. Organic NPs: (a) liposome; (b) polymeric micelle; (c) polymersome; (d) polymeric nanoparticle; (e) solid lipid nanoparticle. Inorganic NPs: (a) mesoporous silica; (b) gold particle; (c) iron particle; (d) hydrogel. Extracellular Vesicles: (a) microvesicle; (b) exosome; (c) apoptotic body. ADCs: (a) internalized ADCs; (b) non-internalized ADCs. TPD-based: (a) PROTAC; (b) LYTAC. There are many categories of DDS, and this diagram shows only some of the typical applications.

Nanocarriers are a new class of tiny carriers, usually less than 200 nm in diameter, with lipid-based nanoparticles, polymer-based nanoparticles and inorganic nanoparticles being the most common nanocarriers (9) (**Figure 2**). Nanoparticles load anticancer molecules by technical means such as physical encapsulation, electrostatic adsorption, and encapsulation, and then enter target cells by cellular endocytosis by binding to specific receptors (20, 21). This results in the effective release of the loaded drug within the target cells. In addition, EVs, which are nanoscale media with a bilayer membrane structure that are shed from cell membranes or secreted by cells, can also be considered nanocarriers. EVs are classified as exosomes, apoptotic vesicles and microvesicles due to different modes of origin and diameter sizes (**Figure 2**) (22).However, the methods of loading drugs into EVs differ from those of other nanoparticles, including manipulation of primary cells to overexpress specific substances, physical incorporation of exogenous RNA or protein drugs using electroporation, or chemical treatment of the cells themselves (23, 24). In addition to entering the target cell by endocytosis or ligand-receptor binding, EVs can fuse directly with the plasma membrane of the target cell and release the loaded drug (25).

According to early research, nanoparticles have been widely developed for delivering chemotherapeutic drugs to tumors, but fewer for tumor immunotherapy. To date, 11 nanocarrier drugs have been approved and marketed for the treatment of solid tumors (**Table 1**), including breast cancer, ovarian cancer, lung cancer, liver cancer, *etc* (15, 26). Nevertheless, most of these drugs are chemotherapy-based, except for Mepact, made from liposome-encapsulated mifamurtide, which is used for immunotherapy of osteosarcoma. In the last two decades, continuous advances in nanoengineering technologies and progress in understanding the importance of nanoparticle properties (*e.g*., size, shape, and surface properties) on biological interactions have created new opportunities for developing nanocarriers for tumor immunotherapy applications. Notably, continuously improving nanodrug delivery systems can enhance antitumor immunotherapy efficacy through a variety of advantages, including improving drug solubility, targeting drug delivery, overcoming physiological barriers to drug delivery, and providing a multifunctional drug delivery platform (27). In the following, we will focus on the latest research on the use of nanoparticles-based DDS for cancer immunotherapy under different immune mechanisms.

### 2.1 DDS for activating dendritic cells

Dendritic cells (DCs) are the most potent antigen-presenting cells (APCs) in the body, involved in antigen recognition, processing and presentation, effectively stimulating T cell responses and inducing the production of specific cytotoxic T lymphocytes, thereby initiating, maintaining, and regulating the immune response (28). Evidence suggests that impaired DC maturation and inefficient antigen presentation play an essential role in tumor development and progression (28). As a result, various nanoparticles have been developed to promote the activation of DCs to initiate and enhance the anti-tumor immune response.

#### 2.1.1 DDS for delivering STING agonists

The cGAMP-STING immune signaling pathway plays a very important role in the body’s anti-tumor and anti-infection processes (29). In the mammalian natural immune system, cytoplasmic DNA derived from tumors or microorganisms activates cyclic GMP-AMP synthase (cGAS) to synthesize the second messenger 2’, 3’-cyclic guanosine monophosphate adenosine monophosphate (cGAMP). cGAMP binds to stimulator of interferon genes (STING) causing it to form a dimer, recruiting TANK-binding kinase 1 (TBK1), phosphorylating and activating interferon regulatory factor 3 (IRF3), induces the expression of type I interferon (IFN) and other cytokines in DCs (29). Matured and activated DCs subsequently promote antitumor immunity by inducing tumor antigen specific CD8+ T cells in lymph nodes (30–32). This indicates that STING agonists have great potential in the field of anti-tumor immunotherapy.

As cyclic dinucleotides (CDNs), the natural ligands of STING, are small hydrophilic molecules, impermeable to membranes and susceptible to rapid degradation by nucleases, they are not suitable as reagents for systemic administration, and the researchers recommend intra-tumor injection as a delivery method (32). Intratumoral administration of STING agonists induces local T cell activation, with significant inhibition of proximal but not distal tumors (32). Consequently, for the translation of STING agonists into clinical use, it is not sufficient to rely solely on intratumoral injection as a modality. To achieve higher rates of tumor control, researchers have developed several liposomes and polymer-based nanocarriers for systemic delivery of STING agonists (33–36). For example, Ning Cheng et al. constructed liposome-based nanoparticles and Mohamed Wehbe et al. encapsulated CDNs by using endosome-destabilizing polymer vesicles. The use of these nanoparticals significantly extended the elimination of half-life of CDNs, leading to the increased tumor accumulation and consequently increased STING activation in the tumor microenvironment (TME) (35, 36). However, due to their limited ability to spread through the dense tumor extracellular matrix, these strategies result in only a tiny proportion of cancer cells or tumor-infiltrating immune cells taking up CDNs (35, 36).

After years of exploration, researchers believe that in addition to the size of the nanoparticle and the choice of surface properties, the nanoparticle’s shape is a critical factor in drug cycle time, biodistribution and cellular uptake to achieve more effective drug delivery (37, 38). Most of the nanoparticles that have been approved for marketing and are under development are produced in spheres, such as liposomes, gold nanoparticles, mesoporous silica, *etc* (15, 26). However, Discher and Arnida et al. found that rod-shaped nanoparticles showed reduced clearance and longer circulation times in mice than spherical nanoparticles with the same surface properties (39, 40). This suggests that it may be possible to improve the delivery efficiency of nanoparticles by changing their shape. Eric L. Dane et al. demonstrated a non-spherical lipid nanoparticle (41). Based on the design concept of antibody-coupled drugs, the researchers bound the CDNs pre-drugs to polyethene glycolized lipids via cleavable linkers. They doped them into lipid nanodiscs, forming LND-CDNs (referred to later as LNDs). In contrast to the uniform spherical vesicle shape of liposomes, lipid nanodiscs are disc-shaped nanoparticles formed by self-assembly. They found that LNDs were more readily absorbed by cells or penetrated tumor spheres, while releasing more parental CDNs, due to the ability of LNDs to deform and enter pores smaller than their equilibrium diameter fully. This result was verified in the MC38 tumor-bearing mice after intravenous administration. At the same time, LNDs showed a 0.6-fold prolonged circulating half-life compared to liposomes. In conclusion, LNDs have a clear advantage over conventional liposomes in delivering STING agonists deep into the tumor. Following intravenous administration of LNDs and conventional liposomes to MC38 tumor-bearing mice, LNDs accumulated twice as much in CD11c+ DCs as conventional liposomes, were taken up by more tumor cells, showed higher levels of IFN-β in tumor tissue and induced massive tumor cell death. In conclusion, these results further demonstrate the importance of nanoparticle shape in improving delivery efficiency.

Except for changing the physical properties of the carrier, scientists have also developed active targeting strategies to improve the delivery efficiency of STING agonists (42). After activation of the STING pathway, type I IFN was reported to induce CD103+ DCs to produce the chemokines CXCL9 and CXCL10, thereby recruiting T cells to the tumor (30, 43). Clec9a is a C-type lectin receptor, which is highly expressed in CD8 α + and CD103+ dendritic cells, and not expressed in any other hematopoietic cells (44, 45). Aatman S. Doshi et al. used Clec9a peptides targeting CD103+ DCs to modify liposome agents encapsulated with CDN (Adu-S100) (42). Meanwhile, they used the same method to prepare non-targeted liposomes. In MC38 tumor-bearing mice, both liposomes carrying STING agonists could promote the uptake of STING agonists by CD103+DCs, lead to the activation of APCs, and significantly enhance the infiltration of CD8+T cells in tumor. However, higher levels of type I IFNs and IL-6 were induced in the targeted group than in the non-targeted group. This confirms the advantages of targeting Clec9a strategies. Beyond this, they found that the tumor-bearing mice showed a robust immune response even after intravenous administration at a low dose of 0.1 mg/kg. In addition to its use as monotherapy, the compound also showed significant anti-tumor activity when combined with PD-L1 antibody. Surprisingly, effective immune stimulation could also be achieved after systemic administration of targeted liposome drugs in tumors with relatively low cytotoxic immune cell infiltration, such as B16F10.

Apart from lipid nanoparticles, VLPs have also been developed as carriers for delivering STING agonists (46). VLPs are particulate matter formed by self-assembly of viral capsid proteins, core proteins or envelope proteins, particles that have a similar structure, size and symmetry to the original virus but lack the genome and replication enzymes and cannot replicate autonomously (47). VLPs are widely used in developing prophylactic and therapeutic vaccines for various diseases, including solid tumors (48–50). Recently, Eric L. Dane et al. reported using virus-like particles (VLPs) to deliver the STING agonist 2′3′-cGAMP (46). As a natural mammalian STING agonist, 2’3’-cGAMP has been reported to activate STING in DCs immediately after fusion by being packaged in enveloped virus particles (51, 52). The VLPs synthesized by EricL.Dane et al. to encapsulate cGAMP consist of HIV-1 structural protein and vesicular stomatitis virus glycoprotein envelope glycoprotein. This study showed that cGAMP-VLPs were approximately fifty times more efficient than conventional liposomes at delivering cGAMP into cells. Different from the previous described studies, the team focused on the effect of intratumoral injection on tumor in mice. After intratumoral injection of cGAMP-VLP, the subcutaneous tumors of MB49 tumor-bearing mice were eliminated. And CD4+T cells in the blood of some mice treated with cGAMP-VLP increased significantly, while CD8+T cells in the other mice increased significantly. In the B16-OVA double tumors mice, there was an increase in CD8+ T cells and a decrease in Tregs and NK cells on the side of the tumor which was intratumoral injected with cGAMP-VLP. At the same time, CD8+T cells increased in distal tumors, while Tregs and NK cells did not change significantly. Importantly, cGAMP-VLP can significantly inhibit local and distal tumor growth and has a synergistic effect with anti-PD1. The combination with VLPs can effectively make up for the defect of local injection of free STING agonist. In conclusion, this targeted delivery of STING agonists via a vector-based approach is an up-and-coming therapeutic option for improving anti-tumor immune responses in patients.

#### 2.1.2 DDS for delivering DC vaccine

Active immunity is antigen stimulation to induce the body to produce antibodies. In the case of cancer immunotherapy, vaccination is a form of active immunotherapy. In recent years, numerous clinical trials have been initiated using therapeutic DC vaccines to boost active immunity and in combination with ICIs (53, 54). Based on this, various new nano-delivery systems have been used to develop and design targeted DCs, including lipid-based, polymeric, inorganic, EVs and other nanoparticles (55–58), among which liposome-based tumor vaccines have been studied in clinical trials (59–61). Compared to other nano-delivery systems, EVs have very high biocompatibility, are effective in avoiding clearance by the mononuclear phagocyte system and are relatively safe after degradation by lysosomes in recipient cells upon completion of the delivery task (62). This section reviews the latest research on the use of EVs for DC vaccine development.

Exosomes are extracellular vesicles with diameters ranging from about 30 to 150nm, which are formed by inner body limiting membrane sprouting inward (63, 64). Exosomes act as important messengers of intercellular communication, and their ability to transport biomolecules to recipient cells makes them very attractive in drug delivery (65). Tumor-derived exosomes (as Tex for short in this study)contain many of immunomodulatory proteins, and co-stimulatory and adhesion molecules and are considered promising candidates for tumor vaccines (66). However, there are also limitations, such as poor immunogenicity and the possibility of promoting tumor development (67). Ongoing studies have modified Tex, for example by encapsulating immune agonists or adjuvants, or by engineering surface modifications to overcome these shortcomings (67, 68). For instance, Lanxiang Huang et al. developed a Tex vaccine derived from an α-lactalbumin (α-LA)-overexpressing breast cancer cell line. They encapsulated Hiltonol (toll-like receptor 3 agonist as a typical immune adjuvant) and human neutrophil elastase (ELANE, an immunogenic cell death inducer) inside the Tex, named Hiltonol-ELANE-α-LA-engineered exosomes (HELA-Exos), as an *in-situ* DC vaccine for the treatment of breast cancer (68). It has been reported that α-LA is specifically expressed in most human breast cancers. It is worth mentioning that immunogenic cell death (ICD) is an important way to activate the immune system against cancer (69). ICD refers to the process that when tumor cells die under external stimulation, they change from non-immunogenicity to immunogenicity and release a series of signal molecules to mediate the anti-tumor immune response (70). It showed potent antitumor activity after intravenous administration in an *in situ* triple-negative breast cancer (TNBC) mouse models and human breast cancer-like organs. HELA-Exos carries an ICD inducer that promotes tumor antigen exposure and immunostimulant release, favoring enhanced uptake of dying tumor cells by DCs (71, 72). Due to the significant expression of toll-like receptor (TLR) 3 in DCs, the combined effect of TLR3 agonist and ICD induction allowed HELA-Exos to induce DCs to mature more effectively and had a more significant triggering effect on CD8+T cells. For tumors with dense fibrotic stroma, such as pancreatic ductal adenocarcinoma (PDAC), it is difficult for conventional drugs to break through obstacles (73). In order to effectively promote the maturation of DCs in PDAC, Wenxi Zhou et al. developed an exosomal vaccine targeting DCs containing CCL22 siRNA derived from mitoxantrone (MTX)-treated PDAC cells, called spMEXO (74). MTX can induce ICD in tumor cells, and exosomal membranes derived from MTX-treated PDAC cells can express immunostimulatory signals that promote the maturation of DCs (75). To generate the production of more tumor-specific T cells, they added melanoma antigen recognized by T-cells 1 (MART-1) peptide to the surface of exosomes. The MART-1 peptide has been reported to be a very common immunogenic epitope of HLA-A2-restricted melanoma-specific tumor-infiltrating lymphocytes with the ability to expand CD8+ T cells (76). In an *in situ* PDAC mouse models, spMEXO could exert potential therapeutic effects by promoting the maturation of DCs, activation of CTLs, secretion of anti-tumor cytokines and blocking recruitment of Tregs by DCs through the CCR4/CCL22 axis, successfully inhibiting tumor growth. In addition, given the hypovascularisation and hypoperfused vessels in PDAC, they concluded that using intramuscular administration to transport the drug through the lymphatic circulation was a better option. They compared the tumor targeting efficacy of intravenous and intramuscular administration and showed that spMEXO accumulated at the tumor site 2.8 times more after intramuscular than intravenous administration. This suggests that the optimal route of administration should be chosen according to the vascular differentiation of the tumor.

For the present, subcutaneous or intramuscular injection of tumor vaccine is used in most clinical trials (77, 78). Compared with injecting vaccines, oral vaccines can activate mucosal immunity and facilitate vaccination (77, 79). Over the past two decades, the anti-tumor effects of several oral vaccines based on carriers such as liposomes, polymeric nanoparticles and oil droplets have been unsatisfactory due to the complex environment of the gastrointestinal tract and the presence of intestinal epithelial barriers (77, 80, 81). Professor Guangjun Nie’s team at the National Nano Centre of China proposed using genetically engineered E. coli-derived outer membrane vesicles (OMVs) as carriers for oral tumor vaccines targeting DCs (82). OMVs can penetrate the intestinal epithelial barrier and interact with immune cells in the intestinal lamina propria, which are recognized explicitly by DCs to activate immune responses, making them a promising carrier for oral tumor vaccines (83). The team used fusion protein binding to fuse tumor antigen and the Fc fragment of mouse immunoglobulin G to the C-terminus of ClyA, the surface protein of OMVs (ClyA-Ag-mFC) (82). Considering that prolonged antigenic stimulation leads to immune tolerance of the organism, they introduced arabinose (Ara) inducible promoter to modify E. coli so that the expression of the fusion protein on its secreted OMVs was controlled by the Ara inducible promoter. The simultaneous oral administration of Ara and the modified OMVs allowed the controlled *in situ* production of a tumor vaccine, OMV-Ag-mFC, in the intestine. In MC38 tumor-bearing mice, oral administration of OMV-Ag-mFC significantly improved the tumor-suppressing microenvironment: tumor-infiltrating CD3+ T cells, CD3+CD8+ T cells, CD3+CD4+ T cells and CD11c+ DCs were increased, and immunosuppressive CD3+CD4+CD25+ Tregs were suppressed considerably. More importantly, the oral ClyA-OVA-mFc vaccine induced long-term adequate immune memory in healthy mice. Using the concept of vector and adjuvant integration, the team has been working for a long time to develop and study nano-tumor vaccines based on bacterial membrane materials and has achieved several groundbreaking results. Related research can be found in a review published by the team in 2022 (84).

### 2.2 DDS for promoting the polarization of tumor-associated macrophages from M2 to M1

Tumor-associated macrophages (TAMs) constitute one of the largest populations of tumor-infiltrating immune cells, up to 50% of all cells in some solid tumors and are the main drivers of the tumor microenvironment (85). TAMs with high plasticity and heterogeneity are usually repolarized by different stimulatory factors into two opposite phenotypes: M1-like TAMs with anti-tumor activity and M2-like TAMs that suppress T-cell function (86). Therefore, effective targeted delivery of drugs that modulate polarization to TAMs is a promising strategy to improve the immunosuppressive tumor microenvironment.

As a traditionally approved anti-inflammatory drug, salicylic acid and its derivatives reprogram TAMs from M2 to M1 type by inhibiting cyclooxygenase-2 (COX-2) and its downstream product prostaglandin E2 (PGE2) (87, 88). To better validate the novel role of salicylic acid in anticancer therapy, for the first time, Kai Sun et al. reported a hypoxia stimuli-responsive iron-5,5’-azosalicylic acid nanoscale coordination polymer nanodrugs (FeNCPs) (89). Afterwards, they coupled polyethylene glycol (PEG) on the surface of FeNCPs. The PEG modification was reported to have the advantages of prolonged half-life, reduced, or disappeared immunogenicity, as well as reduced toxic side effects (90). PEGylation of nanoparticle surfaces is a widely used strategy in the pharmaceutical field (90). Hypoxia is a typical feature of solid tumors, which can promote the over-expression of azo reductase (91). In the 4T1 tumor-bearing mouse models of intravenous injection of PEG-FeNCPs, PEG-FeNCPs could be degraded by azo reductase under hypoxic conditions to release 5-aminosalicylic acid. 5-aminosalicylic acid was able to alleviate immunosuppressive tumor microenvironment and enhance the immune response by redirecting TAMs to the M1 phenotype. Notably, iron-based nanomaterials have also been reported to convert TAMs to the M1 phenotype (92). The results of the study showed the maturity of DCs in tumor draining lymph nodes, the infiltration of CD8+ cytotoxic T lymphocytes and the proportion of CD80+ M1-like TAMs were significantly increased, while CD206+ M2-like TAMs was on the contrary. More importantly, PEG-FeNCPs significantly inhibited the growth of tumor in mice.

Metformin (Met) is well known as a standard drug for treating diabetes mellitus. In recent years, newly discovered anti-tumor effects have made Met another hot topic in “new uses of old drugs”. Numerous studies have shown that Met can regulate tumor metabolism, inhibit tumor cell proliferation, migration, and invasion, and induce apoptosis (93, 94). More importantly, Met can exert anti-tumor effects by ameliorating hypoxia, chronic inflammation, and the immunosuppressive microenvironment (95). Recent studies have shown that met significantly downregulates M2 TAM markers and shifts the polarization of TAMs from M2 to M1, thereby inhibiting the pro-tumor effects of M2 TAMs (96, 97). Zhaohan Wei et al. developed mannose-modified murine macrophage-derived microparticles (Man-MPs) loaded with Met, termed Met@Man-MPs (98). Cell microparticles (MPs) are extracellular vesicles with a diameter of 100-1000nm that fall off by cells in response to various endogenous or exogenous stimuli (22, 99). Like exosomes, MPs has the characteristics of transferring messenger molecules, enzymes, and nucleic acids between cells, and it is a drug carrier with great potential (100). MPs derived from macrophages has natural tumor targeting ability (101). Mannose modification enables Met@Man-MPS to target M2 TAMs, as the mannose receptor is highly expressed on M2 TAMs. In H22 tumor-bearing mice, Met@MPs significantly enhanced the protein expression of CD80 and CD86 (M1-related markers), while decreased the mRNA expression of Arg1, Mrc1 and Mgl1, and protein expression of CD206 (M2-related markers).Meanwhile, the targeted macrophages effectively reconstituted the anti-tumor immune microenvironment by reducing the number of Tregs and MDSCs in tumor tissue and increasing the recruitment of CD8+ T cells. Moreover, Met@Man-MPs remarkably inhibited tumor growth and prolonged the survival time of mice. Surprisingly, when combined with anti-PD-1 antibodies, Met@Man-MPs effectively induced the degradation of tumor-associated extracellular matrix type I collagen, thereby enhancing the accumulation of anti-PD-1 antibodies in tumors and their anti-cancer activity.

### 2.3 DDS for delivering ICD inducers, turning “cold” tumors into “hot” tumors

Based on the space distribution of cytotoxic immune cells in tumor microenviron-ment, tumors are divided into three basic immunophenotypes: immune-inflamed, immune-excluded, and immune-desert phenotype (102). Immuno- inflamed phenotypic tumors are called “hot tumors” due to high T cell infiltration, increased IFN-γ signal transduction, expression of PD-L1 and other reasons (103). And this kind of tumors are often more sensitive to immunotherapy (104, 105). The so-called “cold tumors” are immune-excluded and immune-desert phenotypic tumors (106). Cold tumors are characterized by poor T cell infiltration, low mutation load and low PD-L1 expression (106). Poor T-cell infiltration in “cold” tumors is a major cause of poor immune response, and researchers typically induce ICD to promote the recruitment and activation of tumor-specific T cells to “ignite” the tumor and improve the efficacy of immunotherapy (107). Doxorubicin (DOX), a classic ICD inducer, alone is not sufficient to activate a strong immune response to ignite cold tumors and therefore needs to be combined with other immunotherapies. Pengkai Wu et al. synthesized novel carrier-free fluorinated polymer nanoparticles based on DOX, siTOX and melittin, called FD/FM@siTOX NPs (108). Thymocyte selection-associated high mobility group box protein (TOX) is a key regulator of T cell failure (109). They combined DOX with siRNAs that inhibit TOX expression to reduce T-cell depletion while increasing T-cell infiltration through induction of ICD. Notably, melittin can selectively induce tumor cell death, and their membrane solubilizing properties allow for safe and effective siRNA transfection (110, 111). In the 4T1 tumor-bearing mouse models, it was confirmed that FD/FM@siTOXNPs had significant advantages in enhancing the effect of ICD, significantly increasing the expression of cytokines perforin (PRF), IFN- γ and granzyme-B in CD8+T cells and inhibiting the depletion of CD8+T cells. Intravenous injection of FD/FM@siTOXNPs can significantly inhibit tumor growth and liver metastasis in mice and prolong the survival time of mice. In conclusion, this combination strategy can enhance the overall anti-tumor immune response of FD/FM@siTOX NPs and is a promising DDS to transform “cold” tumors into “hot” ones.

During immunotherapy, activated CD8+ T cells can mediate tumor cell death by secreting PRF to punch holes in the cell membrane (112). However, the membrane repair process may be a key reason for immunotherapy failure, especially in cold tumors with insufficient infiltrating CD8+ T cells (113, 114). Zhanwei Zhou et al. suggested that supplementation with exogenous cytotoxic proteins prior to pore closure may enhance CD8+ T cell-mediated tumor cell killing (114). They injected human serum albumin nanoparticles containing the photothermal agent IR780 (HIR780), ribonuclease A (RNase A), poloxamer 407 and α-cyclodextrin (the primary material for hydrogel formation) into the tumor for *in situ* gelling (*in situ* gelling is the administration of a drug in a solution form with an immediate phase transition at the site of administration from a liquid state to a non-chemically cross-linked semi-solid formulation of the gel). In the 4T1 tumor-bearing mice, a mild photothermal effect at 45°C can induce ICD and activate an immune response, thereby activating CD8+ T cells to secrete PFR to pierce the plasma membrane of tumor cells, promoting RNase A entry into tumor cells, and activating caspase-3 and gasdermin-E pathways to induce apoptosis and scorch death to activate immunity further. CD8+T cells infiltrated efficiently in the tumor tissue, resulting in a significant up-regulation of IFN- γ expression. Without suspense, the mouse tumor was significantly suppressed. However, according to previous studies, mild photothermal therapy (PTT) can induce upregulation of PD-L1 on tumor cells and protect tumor cells from CD8+ T cell attack (115). Therefore, it is necessary to add a PD-L1 antibody to the above combination of drugs. This research team conducted a study on the simultaneous delivery of HIR780, RNase A and PD-L1 antibodies. It showed that this hydrogel formulation containing PD-L1 antibodies had significantly stronger anti-tumor effects than the formulation without PD-L1 antibodies (114).

Photodynamic therapy (PDT) is also a kind of immunotherapy to enhance the anti-tumor effect by inducing ICD (116). PDT means that with the participation of oxygen molecules, the photosensitizer is irradiated by an excited light source to produce cytotoxic reactive oxygen species (ROS), which induces ICD and leads to the death of a large number of tumor cells (117). However, most photosensitizers are prone to aggregation due to their poor solubility in the biological environment, and the quenching effect induced by molecular assembly further reduces the fluorescence emission and ROS production ability of photosensitizers (118). To address this issue, Hongmei Cao et al. developed a mitochondria-targeted photosensitizer with aggregation-induced emission (AIE) properties, namely MBPN-TCyP (119). Their previous studies have shown that encapsulation of AIE photosensitizers with amphiphilic polymers not only results in unsatisfactory loading, but also limits the intramolecular movement of the photosensitizer, leading to inefficient mitochondrial targeting (120). Therefore, in the current study, they used DCs-derived EVs (DEVs) as delivery carriers, and this bionic packaging resulted in excellent biocompatibility of the AIE photosensitizer, which effectively avoided clearance by the mononuclear phagocyte system, thereby specifically targeting the mitochondria of tumor cells and generating large amounts of ROS (119). In 4T1, CT26 tumor-bearing mice, the mitochondrial targeting ability allowed DEV-AIE to induce a better ICD response. Furthermore, DEV-AIE could directly activate T cells via natural ligands (CD80/86, MHC I/II) on the DEV membrane. The percentage of activated CD4+ T cells and CD8+ T cells was significantly increased in tumor tissues treated with DEV-AIE. Similarly, the tumors in mice were significantly inhibited.

In conclusion, compared with conventional drugs, nanocarrier-based DDS have significant advantages, in terms of protection of drug molecules and considerable improvement of drug stability; specific targeting of drug delivery after processing and modification; delayed drug release time, thus prolonging its duration of action; and to some extent, improved drug absorption and utilization.

Unfortunately, however, almost all of these success cases have only been achieved in animals and were rarely used in clinical trials. Nanodrug delivery systems still face significant challenges in clinical translation (121, 122). Due to the diversity of materials, we divided the nanoparticles into different subcategories to show their respective advantages and disadvantages (**Table 3**). On the other hand, nanoparticles also face unique challenges related to their biological, technological, and clinical limitations. One of the main problems in clinical transformation of nanoparticles is the difference between preclinical studies in animal models and clinical studies in humans (123, 124). An example of this is the enhanced permeability and retention (EPR) effect, which refers to the phenomenon that some macromolecular substances of a specific size (such as nanoparticles and some macromolecular drugs) are easier to penetrate tumor tissue and remain for a long time than normal tissues (123). This has been verified in animal models, but there is a lack of reliable evidence in humans (123). The problem of disease heterogeneity in different patients in different periods is also a major challenge in the clinical transformation of nanodrugs. In terms of technical synthesis, there must be a consistent and highly repeatable drug synthesis formula before entering clinical application. However, most preclinical experiments use small quantities of synthesized nanoparticles, and it is not certain that the same results can be obtained after large-scale production (125, 126).

**Table 3:** Advantages and limitations of different drug delivery systems.

Classification of DDS		Advantages	Limitations
**NPs**	**Polymer nanoparticle**	Well-balanced biocompatibility and biodegradability; high therapeutic drug loading; enables active targeting and intelligent response	Easily binds to negatively charged non-specific cells or proteins; some materials are highly cytotoxic; low efficiency of gene transfection
	**Polymer micelle**	Prolonged retention in body; well tissue permeable and biocompatible; capable of being degraded in the body; structures are easily modified; have a special “core-shell” structure	Prone to leakage and sudden release of drugs due to poor physical stability
	**Liposome**	Excellent biocompatibility、 bioavailability and safety; wide range of indications for the drugs contained	Less stabilized; low drug loading rate; the phospholipids contained are easily oxidized; vulnerable to metals, radiation, heat, PH, and enzymes
	**Inorganic nanoparticle**	Simple preparation and low toxicity allow for large-scale use	Metal NPs are less biocompatible; non-metallic NPs are poorly loaded and tend to agglomerate
	**Extracellular vesicle**	Low immunogenicity; natural targeting; very highly biocompatible and effective in avoiding clearance by the mononuclear phagocyte system	Unable to produce on a large scale due to technical constraints; stricter conditions of transportation and storage; long-term storage alters biological activity
	**NPs from other materials**	Ability to integrate multiple therapeutic agents, fluorescent moieties and targeting ligands; higher affinity for binding to sites, better targeting	Lower transfection rate of biomolecule vectors; complex nanoparticle structure poses more risks to biosafety
**ADC**		Reduce systemic side effects of toxic drugs due to strong targeting; high toxin activity	Difficult to develop due to high technical barriers; tend to agglomerate resulting in a decrease in its ability to bind antigens;
**PDC**		Smaller molecular weight for better tissue penetration; low intrinsic immunogenicity; easily synthesized and less costly; easier to improve drug stability by structural modification	Poor cyclic stability; low oral bioavailability; lower tissue specificity and tumor targeting of peptides compared to monoclonal antibodies
**TPD**		Broader scope of action, higher activity, targeting of “non-druggable” targets; higher selectivity, activity and safety compared to traditional small molecule inhibitors; overcoming resistance to traditional medicines	Poor permeability and oral bioavailability due to higher molecular weights; due to technical limitations, the ability to degrade target proteins cannot be assessed quickly and in large quantities; mechanisms of action are still not clear enough

As unique natural nanoparticles, the complex mechanism of delivery therapy mediated by EVs in human body is largely unknown (64, 127). For example, will the membrane structure and other physical and chemical configurations of EVs affect their tumor tendency? What is the applicability of different cargo loading methods for wrapping different antineoplastic compounds? Will tumor-derived exosomes promote tumor progression through some mechanism? Therefore, it is vital to solve our knowledge gap for improving EVs manufacturing. In addition, since the lack of standardized separation and purification scheme, the large-scale production of EVs is also an urgent problem (128). The low load of therapeutic goods in EVs is also the main challenge for its application in targeted therapy (129). This may be because EVs contains part of the contents of the mother cell in the process of formation (129). In short, although there are major challenges and difficulties in the application of nanoparticles-based drug delivery systems, they show great potential in the field of biomedicine for advanced drug delivery and treatment.

## 3 DDS based on coupling technology for cancer immunotherapy

The systemic toxicity of chemotherapeutic drugs has been a challenge, and the use of coupling technology has enabled the targeted delivery of chemotherapeutic drugs, effectively reducing their systemic toxicity (10, 130, 131). Currently, ADCs and peptide-drug conjugates (PDCs) are the two most widely developed coupled drug delivery systems. They are carrier-independent molecular drug delivery systems. Unlike the nanocarrier-based drug delivery systems described above, they combine the specific targeting properties of monoclonal antibodies (mAbs) or peptides with the antitumor properties of cytotoxic molecules to selectively deliver chemotherapeutic drugs to tumor tissue, thereby reducing systemic toxicity. Other than the technological updating of the original delivery systems, scientists have continued to develop new delivery concepts such as targeted protein degradation (TPD), which include proteolysis targeting chimaeras (PROTACs), lysosome targeting chimaeras (LYTACs), and others (132–134). In the following, we will focus on the characteristics of ADCs, PDCs and TPD technologies and the latest research on their application in cancer immunotherapy.

### 3.1 Antibody-drug conjugates

In the early 20th century, Paul Ehrlich, the German scientist who founded chemotherapy, first hypothesized that if toxic drugs were mounted on carriers that specifically target tumor cells, it might be possible to precisely kill cancer cells without harming normal cells (135). Research has gradually shown that some antibodies can recognize tumor cell-surface antigens (tumor specific antigen, TSA; tumor-associated antigen, TAA) and thus target tumor cells (136). The concept of ADCs was first proposed in 1967, but research remained at a theoretical level due to technical limitations. Since 1975, the development of hybridoma technology has enabled the mass production of monoclonal antibodies (mAbs). In recent decades, an increasing number of mAbs have been approved for treating various solid tumors, such as trastuzumab, rituximab and cetuximab (137–140). However, when used as single agents, mAbs are not as effective as chemotherapeutic agents in killing cancer cells (141). Therefore, ADCs have been widely investigated as novel agents that can compensate for the deficiencies of mAbs and cytotoxic drugs.

ADCs have three main components: mAbs that recognize tumor cell surface antigens, payloads of cytotoxic drugs and linkers that connect the two, as shown in **Figure 2** (130). The linker is a crucial component that prevents the dissociation of ADCs in the bloodstream. Most ADCs are internalizable, and drug release can be divided into two modes depending on whether the linker is cleavable (130, 142). Cleavable linkers depend on the physiological environment within the tumor cell, such as low pH, protease hydrolysis or high intracellular glutathione concentrations, to release their ADC carrier payload. If the linker is not cleavable, the mAbs are degraded in the lysosome, leaving the linker attached to the cytotoxic drug for release into the cytoplasm. This non-cleavable linker is more stable in the blood and has relatively low off-target toxicity (143).

In addition to acting by delivering cytotoxic drugs to the tumor site, the antitumor effect of some ADCs involves the function of the Fc fragment in the mAb molecule (144, 145). These ADCs can not only recognize the target specifically through its Fab domain, but also exert its immune effect function through Fc fragment. Antibody-dependent cell-mediated cytotoxicity (ADCC), antibody-dependent cell phagocytosis (ADPC), and complement-dependent cytotoxicity (CDC) are the most important effector modes in the Fc fragments (145). Fc fragments bind to the FcR on the surface of killer cells (NK cells, macrophages, *etc.*), enabling ADCs to directly kill tumor cells. And we refer readers interested in this subject to published articles that specifically discuss the mechanism of effects in Fc fragments (145). Besides, the antibody component of ADCs can specifically bind to epitope antigens of cancer cells and inhibit the downstream signaling of antigen receptors, thereby inhibiting cancer cell growth.

Since the first ADC drug, Mylotarg, was approved by the Food and Drug Administration (FDA) in 2000, eight ADCs have been used clinically to treat solid tumors (**Table 2**) (146). These ADCs, and most of those still in clinical trials, are loaded with cytotoxic drugs, including antitubulin and DNA-damaging agents (14). Some of these cytotoxic drugs, such as anthracyclines, platinum, and ruthenium can induce ICD, leading to activation of DCs, thus, activation of T-cell-mediated anti-tumor immune responses (144). Besides, some ADCs have been reported to lead to increased PD-L1 expression in tumor cells, with synergistic effects between them and immune checkpoint inhibitors (115, 147–149). In addition to traditional cytotoxins, several small molecule immunomodulators have been used in the development of novel ADCs: TLR agonists and STING agonists, known as immunostimulating antibody conjugates (ISACs) (150–152). BDC-1001 is a novel ADC currently in clinical development (Phase I/II, NCT04278144), also known as ISACs (153). It consists of a HER2-targeting antibody and a TLR 7/8 agonist with a non-cleavable linker and is used to treat patients with HER2-positive solid tumors. Other ISACs targeting solid tumors include SBT6050 (Phase I, NCT04460456) and SBT6290 (Phase I/II, NCT05234606) (154, 155). Both drugs use TLR8 agonists as payloads, with the difference that SBT6050 targets HER2 and SBT6290 targets Nectin4 (Nectin cell adhesion molecule 4, a type I membrane protein that is overexpressed in a variety of tumor cells). Their clinical data is worth waiting for.

In recent years, the number of ADCs entering clinical trials has continued to grow because of technological improvements, such as the development of antigen-binding fragments that are more stable in circulation, optimization of the interactions between antibodies and FcγRs, and improved linkers for better plasma stability (156). Unfortunately, some ADCs that have shown enormous potential in preclinical studies have performed poorly in clinical trials. Incomplete internalization is an important reason for the poor clinical application of ADCs. ADCs are often designed to act through internalization mechanisms, as described above. This highly depends on the overexpression of internalizing antigens, which is not a common feature of tumors. Moreover, antibodies are large molecular proteins that diffuse slowly, have limited tumor penetration, and continually bind to cancer cells in the periphery of the tumor near blood vessels, preventing them from penetrating deeper into the tumor (157). Therefore, in the last decade, research has increasingly focused on the development of non-internalizing ADCs with extracellular payload release mechanisms (142, 158). The design and development of non-internalizing ADC does not depend on the internalization process of tumor cells, but on the targeted delivery and release of drugs by targeting non-internalizing antigens on the surface of tumor cells. The linkers of non-internalizing ADCs are designed to be unstable in the extracellular tumor microenvironment and take advantage of the unique chemical or enzymatic environment of tumors compared to healthy tissue to release the payload outside the tumor cells and spread to surrounding cancer cells to exert their cytotoxic effects (142). Currently, the reported targets of non-internalizing ADC mainly include tumor cell membrane proteins and tumor microenvironment (**Table 4**). Due to space constraints, we will not introduce the targets in detail in this article. In a word, compared with the traditional internalizing ADC, the development of non-internalizing ADC obviously expands the range of tumor targets because it does not depend on the expression of highly homologous antigens (142). And, apart from all that, non-internalizing ADC takes advantage of the unique chemical or enzymatic environment of tumor, and will not crack in healthy tissues, so it can reduce toxicity and side effects (142).

**Table 4:** Promising targets for non-internalized ADCs for the treatment of solid tumors.

Classification of targets	Target	Drugs in development (antibody/payload/linker) (reference)
Membrane proteins	PD-L1	**\**
	CEACAM5	Labetuzumab/SN-38/linkers with acid-labile carbonate or ester moieties (159)
	Na^+^/K^+^-ATPase	anti-NKA/anti-dysadherin/polyethylene glycol linkers (160)
Proteins in the extracellular matrix	Gal-3-BP	anti- Gal-3-BP/thiol-containing maytansinoid drug/interchain disulfides (161)
	LRG1	Magacizumab/MMAE/protease-cleavable Val-Cit linkers (162)
	MMP9	anti‐MMP9/broad‐spectrum MMP inhibitor/interchain disulfides (163)
Stroma or tumor vasculature	Collagen	anti- Collagen/SN-38/ester linkage (164)
	Fibrin	anti-Fibrin/SN-38/ester linkage (165)
		anti-Fibrin/MMAE/plasmin-cleavable tripeptide Val-Leu-Lys linker (166)
	Fibronectin	F8-SIP/thiol-containing dolastatins/interchain disulfides (167)
	Tenascin-C	anti- Tenascin-C/MMAE/protease cleavable Val-Cit linker (168)

Whether internalized or not, ADCs has the risk of off-target, which is another important reason for the poor effect of ADCs in clinical application (169). To eliminate the miss effect, researchers mostly mask the antigen binding fragments of the antibody to ensure that the antibody binding characteristics are selectively activated in the tumor site (170). Nonetheless, the function of the Fc domain of the antibody is ignored. Based on this, Adrian Elter et al. proposed an idea that the antibody Fc domain remains inert during circulation and restores the functional properties of the effector when it reaches the malignant tumor (171). They fused the single-stranded variable fragment (scFv) into the C-terminal of the light chain of trastuzumab by matrix metallopeptidase 9 (MMP-9) cleavable linker. ScFv specifically binds the FcγRs interaction site on Fc. Trastuzumab of Fc-masked reached the tumor tissue later, the antibody activated by Fc segment showed full recovery of binding to FcγRs and C1q after MMP-9-mediated splice cleavage and scFv dissociation, as well as restored ADCC and CDC effects. More importantly, the Fc-masked antibody form can produce a synergistic effect with the effector-enhanced engineering antibody. The combination of the two can not only enhance the anti-tumor effect, but also reduce the potential systemic toxicity.

### 3.2 Peptide-drug conjugates

The main components of PDCs include homing peptides, cytotoxic drug payloads and linkers. PDCs have a similar structure to ADCs, except that PDCs use peptides for targeting (131), as shown in **Figure 2**. Homing peptides can be divided into two types: cell-penetrating peptides (CPPs) and cell-targeting peptides (CTPs) (172). CPPs are capable of directly entering cells through different mechanisms and carrying goods in cells (173). CTPs have a good ability to target specific targets, so they have the potential to deliver payloads specifically (172). The use of peptides as targeting agents in drug conjugates has many advantages over ADCs: PDCs have a lower molecular weight, better tissue penetration and very low intrinsic immunogenicity; they are easy to synthesize and less expensive, and it is easier to improve the stability of the drug through structural modifications (174–176). Only one PDC drug has been approved for the treatment of solid tumors, 177Lu-DOTA-[Tyr3]-octreotate (Pepaxto) (133). It was approved by the FDA in 2018 to treat growth inhibitor receptor-positive gastrointestinal pancreatic neuroendocrine tumors. There are many PDCs in preclinical or clinical trials, and we have listed some of the hot global drugs in development for PDCs (**Table 5**).

**Table 5:** PDCs approved for marketing and in clinical trials for the treatment of solid tumors (up to April 2023).

Name	Target	Company	Application	Latest development stage
**Lutathera**	SSTR	Advanced Accelerate Applications	Somatostatin receptor-positive gastroenteropancreatic neuroendocrine neoplasm	Approved (2018.9)
**Pluvicto**	PSMA	Novartis	Castration-resistant prostate cancer	Approved (2022.3)
**Paclitaxel** **trevatide**	LDLR	Shenogen Pharma; AngioChem	Leptomeningeal carcinomatosis, leptomeningeal metastases, brain metastases, HER2-negative breast cancer	Clinical Trial Phase III
**Zoptarelin doxorubicin**	GnRH	Aeterna Zentaris; Tulane University	Endometrial cancer	Clinical Trial Phase III
**EP-100**	GnRH	Esperance Pharmaceuticals	Ovarian cancer	Clinical Trial Phase II
**BT1718**	MMP14	Bicycle Therapeutics	NSCLC, esophageal carcinoma	Clinical Trial Phase I/II
**BT5528**	EphA2	Bicycle Therapeutics	Urothelial Cancer, ovarian cancer, NSCLC, head and neck cancer, TNBC, gastric/upper gastrointestinal cancer	Clinical Trial Phase I/II
**BT8009**	Nectin-4	Bicycle Therapeutics	Bladder cancer, TNBC, NSCLC, ovarian cancer	Clinical Trial Phase I/II
**CBX-12**	Top1	Cybrexa Therapeutics; Exelixis	Solid tumors	Clinical Trial Phase I/II
**PEN-221**	SSTR2	Tarveda Therapeutics	Neuroendocrine neoplasm, SCLC	Clinical Trial Phase I/II
**BGC0228**	Top1; CD44	BrightGene	Solid tumors	Clinical Trial Phase I
**MB1707**	CXCR4	Lirum Therapeutics	Breast cancer; NSCLC, ovarian cancer, pancreatic cancer	Clinical Trial Phase I
**RS-0139**	αvβ3, 5, 6	RS Research	NSCLC	Clinical Trial Phase I
**Sudocetaxel zendusortide**	SORT1	Theratechnologies	TNBC, pancreatic cancer, colorectal cancer, ovarian cancer, endometrial carcinoma, melanoma	Clinical Trial Phase I

Compared to ADCs, PDCs have been less studied as immunomodulators. What is more prominent in this aspect is the PDCs from Bicycle Therapeutics. Based on a fully synthetic restricted bicyclic peptide technology, Bicycle Therapeutics has developed a novel peptide coupling as a tumor-targeted immune cell agonist™ (BicycleTICA™) (177). They link peptides targeting CD137 and tumor-specific receptors, respectively, via a three-armed branched polyethene glycol junction. This conjugate does not contain cytotoxic or immunomodulatory drugs. BT7480 (Phase I/II, NCT05163041) is the first known BicycleTICA™ in this series to enter clinical trials in advanced solid tumors. In preclinical studies with BT7480, Kristen Hurov et al. used spatial proteomics imaging to identify nectin-4+ tumor cells located within the tumor core and surrounded by CD137+ immune cell infiltration in non-small cell lung cancer, head and neck squamous cell carcinoma and bladder cancer. Therefore, they developed PDCs that can target both Nectin-4 and CD137, namely BT7480, which, due to its high affinity for Nectin-4 and CD137, can bring CD137-positive immune cells into contact with Nectin-4-positive tumor cells simultaneously, thereby activating the immune cells to exert anti-tumor effects. In the MC38 tumor-bearing mice expressing nectin-4, intravenous administration of BT7480 promoted CD8+ T cell tumor infiltration and induced complete tumor regression. The compound showed perfect anti-tumor activity and tolerability in rats and non-human primates. BCY12491, the company’s BicycleTICA™, which targets both CD137 and EphA2, has also shown good immunomodulatory and anti-tumor activity (178).

PDCs compensate for some of the shortcomings of ADCs. Still, the short biological half-life of peptides leads to limited distribution and targeting a time of PDCs *in vivo*, limiting the efficiency of payload delivery to tumor cells (131, 179). Because of this shortcoming, the development of PDCs has been relatively slow, and the number of promising drugs developed is much lower than that of ADCs. Although there were some early methods to improve the half-life by chemical modification, the results were not satisfactory (180–183). Because the Fc domain of IgG or albumin in the organism can avoid being degraded by lysosome through the circulatory pathway of neonatal Fc receptor (184–186). In recent years, some researchers have proposed to connect the Fc domain at the C-terminal of the peptide, or to covalently bind the peptide with albumin and recombinant expression it (184, 185). Thus, it can protect the polypeptide and improve its circulation time in the bloodstream. In a nutshell, as more innovative, and improved methods are studied, safer and more effective PDCs for tumor immunotherapy is expected.

### 3.3 Targeted protein degradation

TPD is an important technology that has developed rapidly over the last decade to interfere with the function of target proteins in cancer therapy. Currently the most mature development in this field is PROTAC technology (187). PROTACs are novel protein “degraders” that promote ubiquitination and degradation by delivering E3 ubiquitin ligase ligands to target proteins of interest (POIs). PROTACs consist of three components: ligands that recruit and bind the POI and E3 ubiquitin ligase, respectively, and a linker that connects the two ligands. Unlike conventional therapeutic modalities, PROTACs can degrade the entire POI and eliminate its full function, thus overcoming some potential drug resistance of traditional drugs (187). More importantly, PROTACs can target more targets, including proteins that cannot be targeted by traditional medications (188). The PROTACs technology was first proposed by Professor Craig Crew’s team at Yale University in 2001 (189); in 2008, the team reported the first small molecule PROTACs that successfully targeted the androgen receptor (190); in 2019, the first PROTACs drugs entered human trials: ARV-110 (Phase I/II, NCT03888612), targeting the androgen receptor (AR), for metastatic prostate cancer (128); ARV-471 (Phase I/II, NCT04072952), targeting the estrogen receptor (ER), for ER+/HER2- metastatic breast cancer (191). In phase I clinical trials, ARV-110 reportedly showed promising activity in first line in patients with metastatic desmoplasia-resistant prostate cancer (mCRPC), with prostate specific antigen (PSA) reductions of more than 50% at doses greater than 280 mg (192). And 1 out of 5 patients confirmed partial remission and 80% reduction in tumor size (192). ARV-471 significantly reduced ER expression levels in tumor tissues by a mean of 62% and a maximum of 90% and achieved a clinical benefit rate in 42% of patients (192). Moreover, both had a favorable safety record at all dose levels tested (192). This indicates that PROTAC technology has a very bright future in anti-tumor therapy.

In cancer immunotherapy, immune checkpoint proteins are hot targets for PROTACs. In 2020, Mingxing Hu et al. reported the first PROTAC molecule that effectively degraded IDO1 (193). They designed and synthesized seven IDO1 PROTACs degraders and found that a PROTACs molecule called 2C could induce significant degradation of IDO1, achieving a maximum degradation rate of 93% in Hela cells. In 2021, Yubo Wang et al. developed a PROTACs molecule 21a that could induce intracellular PD-L1 protein degradation in various tumor cells *in vitro* (194). Treatment of MC38 tumor-bearing mouse models with 21a significantly reduced their PD-L1 protein levels, enhanced the toxic effects of CD8+ T cells and inhibited tumor growth. Since PD-L1 is in a continuous cycle of self-renewal from the cytoplasm to the cell membrane, 21a significantly reduced PD-L1 protein levels not only in total but also at the cell membrane.

Due to the limitations of the ubiquitin-proteasome system (UPS), conventional PROTACs act mainly on intracellular proteins, are unable to degrade extracellular proteins and are restricted to membrane proteins (187, 195–197). In addition, there are more than 600 E3 ligases in the human body, and only about 10 of them (CRBN, VHL, IAP, MDM2, DCAF15, DCAF16 and RNF114, *etc.*) have been developed for PROTACs (198, 199). To overcome these problems, more and more PROTACs-derived technologies have been rapidly developed, such as LYTACs (133, 200–202). The concept of LYTACs, first introduced by Dr Carolyn Bertozzi in Nature in 2020, exploits the endocytosis-lysosome pathway to achieve targeted degradation of extracellular and membrane proteins (203). LYTACs consist of two main components, one targeting the lysosome-targeting receptor (LTR) on the cell surface and the other targeting the target protein (antibody, peptide, or another small molecule), linked by a linker. The transport of proteins to the lysosome requires the involvement of LTRs. After the LYTACs molecule forms a complex with the target protein and the LTR, the complex enters the cell by endocytosis and is captured and dissociated by the lysosome, where the target protein is degraded. In contrast, the dissociated LTR is not contaminated and can continue to transport the target protein through the endosomal cycle (204). The most common and well-studied LTRs are the mannose 6-phosphate/IGF-II receptor (M6P/IGF-IIR) and the associated sialic acid glycoprotein receptor (ASGPR), which have been shown to mediate the entry of substrates into the lysosome for degradation as lysosomal transport proteins (205–208).

Although drug development using PROTACs and their derived technologies is very promising and offer new therapeutic avenues for tumor immunotherapy, there are still many challenges to clinical application (209). Apart from the challenges of developing small molecule E3 ligase ligands, developed PROTACs molecules suffer from off-targeting, poor cell permeability, poor cell and tissue selectivity, poor stability, and significant molecular weight (210–213). Beyond, the optimal site for attachment of oligosaccharide structures to antibodies in LYTACs molecules is still undetermined, and this unnatural sugar structure may be highly immunogenic in humans (210). More importantly, LTRs are widely expressed on the surface of most cells, so how to avoid targeting LYTACs to cells that do not represent the target proteins and how to improve the safety of LYTACs are also urgent issues to be addressed (210).

## 4 Other developable areas on DDS for cancer immunotherapy

### 4.1 Cell-based DDS

In recent years cell-based DDS has demonstrated the ability to outperform traditional delivery systems and has been rapidly developing. There are many types of cells that can be used for cellular delivery, including red blood cells, platelets, stem cells, immune cells, and others (214, 215). In terms of tumor immunotherapy, classic cell therapies such as chimeric antigen receptor T (CAR-T) cells, chimeric antigen receptor natural killer (CAR-NK) cells, and T-cell receptor engineered T (TCR-T) cells all work by harvesting the body’s own immune cells, engineering them *in vitro*, and then infusing them back into the body to target and kill tumor cells (216, 217). CAR-T cell therapy is to transfuse autologous T cells into patients after being modified *in vitro* by genetic engineering to treat diseases (216). Up to now, nine kinds of CAR-T cells have been approved for the treatment of hematological tumors (216).

At the same time, there is another popular drug, bispecific T cell engagers (BiTEs), which is used to redirect cytotoxic T cells to their intended tumor targets, just like CAR-T cells (218). BiTEs is a new model of bispecific T cell- redirecting antibodies, which consists of two different single-stranded variable fragments of the antigen binding domain of anti-CD3 and anti-TAA antibodies, covalently linked by small junction peptides (218). T cell immunotherapy based on BiTEs has been successful in several preclinical and clinical trials aimed at hematological tumors, one of which has been approved by FDA for the treatment of leukemia (218).

However, due to the limitation of immunosuppressive microenvironment and serious miss effect, the curative effect of CAR-T or BiTEs alone in solid tumor is not ideal (219, 220). In recent years, studies on the development of nano-drug carrier platforms for these two therapies have been reported, most of which couple modified nanoparticles on the surface of CAR-T cells or nano-antibodies with BiTEs to play a probe-like targeting effect, and these studies have achieved good results in preclinical experiments (221, 222). This potential combination of immunotherapy and DDS will greatly improve the specificity and safety of drugs and bring more hope for the immunotherapy of solid tumors.

### 4.2 Oncolytic virus therapy

Recently, as a new tumor immunotherapy, oncolytic virus therapy (OVT) has aroused great interest. Oncolytic virus (OVs) is a kind of live attenuated virus that can specifically infect and lyse tumor cells without damaging normal cells (223). OVs can be further modified as a vector to selectively deliver therapeutic transgenes to TME, thereby enhancing anti-tumor efficacy or immune response (223). These therapeutic transgenes include costimulatory molecular genes, chemokine genes, cytokine genes and gene sequences of ICIs (224, 225). So far, four oncolytic viruses have been approved for marketing. T-VEC is the first oncolytic virus approved by FDA in 2015 for the treatment of unresectable metastatic melanoma (226). It contains granulocyte-macrophage colony-stimulating factor (GM-CSF) coding sequences that stimulate the immune system. What is encouraging is that in a phase II clinical trial of preoperative T-VEC combined with neoadjuvant chemotherapy, 45.9% of patients achieved complete remission after treatment. And two years after treatment, 89% of the patients had no recurrence. In addition, the selectivity of tumor cells and their ability to induce systemic anti-tumor immune response make OVs a potential immune adjuvant that can enhance the efficacy of other tumor immunotherapy, such as cell therapy and ICIs (225). At present, there are more than 40 early clinical trials exploring the combination therapy of Ovs, PD-L1 monoclonal antibody and CAR-T, and showing good curative effect (225, 226).

## 5 Challenges and prospects

In tumor immunotherapy, DDS are widely used to deliver immune-modulators, tumor vaccines and nucleic acid drugs, and provide an excellent platform for combining immunotherapy with chemotherapy/PTT/PDT, *etc.* Based on different materials and synthetic processes, the various drug delivery systems reviewed in this paper have different characteristics. We summarize their respective advantages and disadvantages, as shown in **Table 3**, hoping to help researchers in the choice of drug delivery strategies. Moreover, due to the diversity of DDS, it is essential to pick the appropriate delivery system according to the characteristics of the target, its distribution, and its biological function (whether it can be internalized or not, *etc.*). Depending on the intracellular localization of target proteins and the characteristics of different cancer types, such as dense tumor stroma and hyper vascularized PDAC, as mentioned above, we should consider different delivery strategies and modes of administration to improve the efficiency of drug delivery and enhance its penetration into the tumor. Another noteworthy issue is the presence of a physiologic barrier as a challenge to be addressed in DDS research (227). Among the methods to enhance penetration, laser-assisted drug penetration has received much attention in the field of pharmacology (228). Besides killing tumor cells by activating photosensitizers in PDT, lasers can also kill tumor cells with heat and disrupt the blood-brain barrier. It has been reported that laser interstitial thermal therapy (LITT) is a popular treatment for glioma in recent years, which can directly stimulate anti-tumor immune response while increasing drug permeability and synergize with systemic immunotherapy (228, 229).

In addition, researchers are constantly searching for more superior DDS combination strategies and have explored that combining nanotechnology with TPD has the potential to produce even more impressive “chemical reactions”. For example, Heng Zhang et al. reported a covalent nanobody-based PROTAC strategy, called GlueTACs, for targeting membrane protein degradation (204). They used highly stable and permeable nanoantibodies that covalently bind to antigens to form complexes, which are subsequently internalized into cells and degraded by lysosomes with the help of cell-penetrating peptides and lysosomal sorting sequences. And it was demonstrated that, compared to PD-L1 blockade, this PD-L1-targeted GlueTACs produced a more robust and longer-lasting PD-L1 degradation effect in the melanoma mouse models. On top of nanobody targeting, a novel photosensitive nanoparticle developed by Ji Qi et al. was able to form multivalent cross-links by binding between PD-L1 on the surface of tumor cells, driving the delivery of endocytosed cross-linking complexes to the lysosome, and acted as a platform of LYTACs to mediate the internalization and degradation of PD-L1 in the lysosome (230).

In addition to existing DDS, researchers continuously explore robust new vectors to deliver drugs to the suitable cells. Recently, Feng Zhang and his team reported that bacterial contractile injection systems (CISs) could be the ‘‘syringe’’ for injecting therapeutic proteins into human cells (231). CISs are remarkably like the contractile tail of T4 phage in that the contractile outer tube uses a substrate complex to form an adhesion point, and the rigid inner tube carrying the therapeutic protein is punctured into the recipient cell by a contractile rotational action to complete protein release. In addition, modification of the tail fibronectin allows CISs to target specific tumor cells. In short, novel drug delivery systems including CISs or more efficient delivery strategies will be gradually explored.

DDS can precisely deliver immunomodulators to target tissues or specific immune cells, thus achieving effective and enhanced immune responses and better therapeutic outcomes. And yet, there are still several outstanding issues that need to be addressed while improving the efficiency of tumor immunotherapy. 1) The requirement of co-delivery of multiple drug classes increases the complexity of carrier design, and the synthesis of complex carriers requires the use of multiple raw materials, each of which needs to be evaluated for safety to reduce the toxicity or off-target effects of the carriers, and the interactions between complex carrier structures and organisms can lead to even more unpredictable biological effects and safety in practical applications. 2) Cytotoxic drugs and immunomodulators usually target different cells, and the co-delivery strategy of DDS usually makes it difficult to achieve precise delivery to different cells in the tumor tissue simultaneously, which may increase the off-target effect of drugs. Therefore, the rational design of DDS with timed and quantitative drug release to achieve precise targeting of different drugs to different cells is a new direction for combination therapy research. 3) Modification of nanoparticles with polyethylene glycol (PEG) is widely considered to be effective means of evading clearance by the reticuloendothelial system and prolonging circulation time (232). Nevertheless, PEGylation is also faced with several controversies, such as hindering cellular interactions, inducing allergic reactions, and stimulating the production of IgM after repeated administration (233). 4) The tumor microenvironment may be highly heterogeneous in diverse patients or in different periods in the patient, which requires researchers to formulate personalized therapeutic regimens at the molecular level. Accordingly, the design of DDS with a simple structure, easy industrial production, good biocompatibility, specific targeting, and high delivery efficiency under the premise of ensuring biosafety is a tough challenge still to be overcome in the current tumor immunotherapy research.

In summary, the current strategy of combining DDS with immunotherapy can significantly improve the efficiency of tumor treatment and reduce systemic toxic side effects and has excellent potential for application. Researchers have used the advantages of diverse types of materials to explore various types of delivery carriers. We have presented as comprehensive a picture as possible of the available DDS in the hope of providing inspiration for clinicians interested in a particular drug carrier. Although there are still some issues yet to be solved, it is believed that, with the further improvement of DDS research, the increasing understanding of materials and the development of new materials, safe and efficient immune drug delivery strategies will be applied to the clinic soon. More cancer patients will be benefited from immunotherapy.

## Author contributions

XL: Writing – original draft, Writing – review & editing. YC: Writing – original draft. YM: Writing – original draft. ZZ: Writing – original draft. DT: Writing – review & editing. YL: Writing – review & editing. XH: Writing – review & editing. TW: Writing – review & editing.
